# Partial FAK suppression promotes tumor growth, an effect reversed by macrophage p110δ PI3K inactivation

**DOI:** 10.1002/1878-0261.70320

**Published:** 2026-09-14

**Authors:** Lydia Xenou, Niki Tzenaki, Anna Tsapara, Maria Tzardi, Evangelia A. Papakonstanti

**Affiliations:** ^1^ Department of Biochemistry, School of Medicine University of Crete Heraklion Greece; ^2^ Department of Pathology University Hospital, School of Medicine, University of Crete Heraklion Greece

**Keywords:** breast cancer, FAK, macrophages, protein dosage effect, therapeutic targeting, tumor growth

## Abstract

FAK/PTK2 is widely considered a therapeutic target in cancer, although its role in tumorigenesis remains context‐dependent. Here, we investigated whether different degrees of FAK suppression produce distinct effects on tumor progression and whether macrophage p110δ PI3K inactivation can counteract adverse effects associated with incomplete FAK inhibition. Using MDA‐MB‐231 breast cancer cell clones stably transfected with FAK/PTK2‐shRNA, we found that strong FAK suppression impaired tumor growth, whereas moderate FAK silencing promoted rapid tumor expansion in female and male mice. Intratumoral FAK/PTK2‐siRNA inducing moderate FAK suppression similarly accelerated breast tumor growth, increased proliferation, reduced apoptosis, and increased circulating tumor cells. Transfer of macrophages expressing genetically inactive p110δ significantly reduced the tumor burden induced by moderately FAK‐silenced breast cancer cells, decreased proliferation, and increased apoptosis. In melanoma tumors, suboptimal pharmacological inhibition of FAK increased tumor burden, whereas combined treatment with a selective p110δ inhibitor prevented this effect, reduced circulating melanoma cells, suppressed proliferation, and enhanced apoptosis. Since FAK inhibitors may not achieve complete and sustained inhibition of FAK activity *in vivo*, our findings highlight the possibility that partial FAK suppression may generate adverse biological outcomes and identify macrophage p110δPI3K as a complementary therapeutic strategy to prevent tumor‐promoting consequences arising from incomplete FAK inhibition.

## Graphical abstract

Partial inhibition of focal adhesion kinase (FAK) can paradoxically promote tumor growth, rather than simply producing a weaker antitumor effect than that observed with strong FAK suppression. In breast cancer and melanoma models, targeting p110δ PI3K, particularly in macrophages, counteracted these tumor‐promoting effects, highlighting the importance of both inhibition intensity and tumor–immune interactions in targeted therapy. Graphical abstract created using chatgpt image generation (openai, 2026) and subsequently edited by the authors.

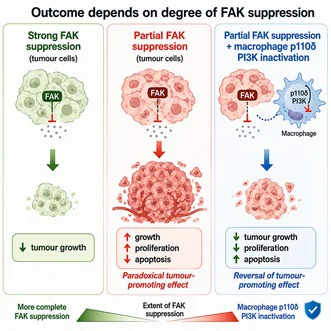

**Abbreviations**
Akt: protein kinase B
ATP: adenosine triphosphate
BrdU: 5‐bromo‐2′‐deoxyuridine
CO_2_
: carbon dioxide
DMEM: Dulbecco's modified Eagle medium
DNA: deoxyribonucleic acid
ERK: extracellular signal‐regulated kinase
FAK: focal adhesion kinase
FCS: fetal calf serum
GFP: green fluorescent protein
GTPase: guanosine triphosphatase
MAPK: mitogen‐activated protein kinase
NC3Rs: National Centre for the Replacement, Refinement and Reduction of Animals in Research
NSG: NOD scid gamma
NT: non‐targeting
p110δ: p110 delta catalytic subunit of phosphoinositide 3‐kinase
pAkt: phosphorylated Akt
PBS: phosphate‐buffered saline
PI3K: phosphoinositide 3‐kinase
PTK2: protein tyrosine kinase 2
PVDF: polyvinylidene fluoride
RRID: Research Resource Identifier
SD: standard deviation
SDS/PAGE: sodium dodecyl sulfate–polyacrylamide gel electrophoresis
SEM: standard error of the mean
shRNA: short hairpin RNA
siRNA: small interfering RNA
STR: short tandem repeat
TUNEL: terminal deoxynucleotidyl transferase dUTP nick‐end labeling
WT: wild type

## 1 Introduction

Breast cancer remains one of the leading causes of cancer‐related mortality in women worldwide [1, 2]. Despite significant advances in diagnosis and treatment, the molecular mechanisms underlying breast cancer progression and metastasis remain incompletely understood [3]. Among the proteins implicated in tumorigenesis, Focal Adhesion Kinase (FAK) has emerged as a central regulator of signals transmitted from integrins and growth factor receptors to intracellular signaling pathways, thereby influencing cell adhesion, migration, proliferation, and survival [4, 5].

FAK is a non‐receptor cytoplasmic tyrosine kinase encoded by the *PTK2 g*ene. Upon integrin engagement or growth factor stimulation, FAK undergoes autophosphorylation at Tyr397, creating a high‐affinity binding site for Src family kinases and initiating downstream signaling cascades [4]. Through interaction with multiple adaptor proteins, FAK modulates several key pathways, including PI3K/Akt, MAPK/ERK, and Rho GTPases [6, 7], all of which regulate cell motility, survival, and cytoskeletal organization [4, 8, 9].

Numerous studies have demonstrated overexpression or hyperactivation of FAK in several cancer types, including breast, colon, prostate, ovarian, and pancreatic cancers. Elevated FAK levels are frequently associated with increased invasive potential, metastatic behavior, and poor clinical outcome [6, 10, 11]. These observations have supported the development of pharmacological inhibitors targeting either the FAK kinase domain or FAK scaffold‐dependent interactions, several of which are currently under evaluation in preclinical and clinical settings [9, 12]. However, the precise role of FAK in cancer biology is complex and highly context‐dependent [9]. Evidence suggests that FAK inhibition may not always produce the expected therapeutic benefits and, in some contexts, may suppress cancer cell proliferation while also altering immune cell function and stromal architecture, thereby reshaping the tumor microenvironment in ways that can paradoxically support tumor resilience to treatment [13, 14, 15]. Importantly, partial modulation of FAK signaling may have biological consequences that differ from those induced by complete FAK ablation, affecting processes such as anoikis resistance, cell polarity, and invasive capacity [16, 17, 18, 19, 20]. Tumor‐associated macrophages are key components of the tumor microenvironment and can promote tumor growth, survival, invasion, and dissemination [21]. Our previous studies have shown that p110δ PI3K, a leukocyte‐enriched PI3K isoform, is implicated in macrophage‐dependent regulation of tumor progression and that macrophage p110δ activity may influence tumor responses to targeted therapies [22, 23, 24].

The present study investigates the effects of differential FAK suppression in the triple‐negative breast cancer cell line MDA‐MB‐231, a model of aggressive breast cancer. To determine how distinct levels of FAK suppression shape cancer growth and progression, we generated clones with moderate (B11) or near‐complete (D1) FAK knockdown and assessed their signaling properties *in vitro* and tumorigenic potential *in vivo* in NSG mice. We further validated the effects of moderate FAK suppression using intratumoral administration of FAK/PTK2 siRNA at a dose that partially reduced FAK expression. We further examined whether tumor‐promoting effects associated with partial FAK suppression could be counteracted by macrophage‐targeted p110δ PI3K inactivation. To extend these findings beyond breast cancer, we also used a melanoma model in which suboptimal pharmacological inhibition of FAK promotes tumor growth and assessed whether combined p110δ PI3K inhibition could prevent this adverse effect. Together, these approaches provide insight into how incomplete FAK inhibition may influence tumor progression and suggest a potential strategy to optimize FAK‐targeted therapies by limiting unintended tumor‐promoting consequences.

## 2 Materials and methods

### 2.1 Cell culture of cancer cell lines

The human breast cancer cell line MDA‐MB‐231 (RRID:CVCL_0062) was kindly provided by Bart Vanhaesebroeck (Ludwig Institute for Cancer Research, London, UK). The B16.F10 (RRID:CVCL_0159) murine metastatic melanoma cell line was a gift from Prof. Papamatheakis (Institute of Molecular Biology and Biotechnology, Crete, Greece). Cell line identity was confirmed within the past 3 years. Cells were authenticated by short tandem repeat (STR) profiling. Cells were cultured in DMEM (Invitrogen, Life Technologies, Waltham, MA, USA) supplemented with 10% fetal calf serum (FCS) and 1% penicillin–streptomycin at 37 °C in a humidified atmosphere containing 5% CO_2_. Cells were routinely tested for mycoplasma contamination and all experiments reported in this study were performed using mycoplasma‐free cells. Prior to injection into mice, cells were cultured to approximately 70% confluence, harvested, counted, and resuspended in sterile PBS.

### 2.2 Plasmid transfection and lentiviral infection‐ construction of stable cell lines

Plasmids carrying constructs for FAK suppression were transformed into *E. coli* to produce recombinant viral particles used for infecting MDA‐MB‐231 cells. The FAK (PTK2) Human shRNA Plasmid Kit (#TR320499) was purchased from Origene (Rockville, MD, USA). The kit uses the pGFP‐C‐shLenti vector, which carries (a) the shRNA expression cassette targeting the gene of interest, (b) a puromycin resistance gene for mammalian cell selection, (c) a tGFP reporter gene for transfection monitoring, and (d) an ampicillin resistance marker for bacterial selection. The kit provides four distinct shRNA constructs (A, B, C, D, and NT) targeting the gene of interest, along with a non‐targeting HuSH 29‐mer scrambled shRNA control vector (NT) to be used as control. Briefly, the procedure was as follows. For plasmid amplification, *E. coli* bacteria were transformed with the five shRNA vectors (A–D and NT) by heat shock, plated on LB–agar containing the appropriate antibiotic, and cultured overnight. Colonies were expanded and plasmid DNA was isolated using a Plasmid DNA purification kit (NucleoBond® XtraMidi Kit from Macherey‐Nagel, Dueren, Germany) according to the manufacturer's protocol. For lentivirus production, HEK293T cells were co‐transfected with the isolated shRNA plasmid vectors together with pVSVG and pΔ8.9 packaging plasmids to produce lentiviral particles. Virus‐containing culture supernatants were collected 24 and 48 h post‐transfection. MDA‐MB‐231 breast cancer cells were transduced with the collected lentivirus to drive their transformation with the respective shRNAs. The efficiency of shRNA incorporation into the tumor cells was monitored using fluorescence microscopy based on GFP expression, and the cell cultures were maintained in puromycin‐containing medium to select successfully transformed cells. Distinct GFP+ cell colonies were expanded, and knockdown efficiency was assessed by western blot analysis of protein lysates. Based on the results of this analysis, different cancer cell lines with different degrees of gene silencing were obtained. Two representative clones were selected: B11 (moderate knockdown) and D1 (almost complete knockdown).

### 2.3 Western blotting analysis

For western blot analysis of total cell lysates, 50–70 μg of protein per sample was resolved by SDS/PAGE and transferred onto PVDF membranes. Membranes were incubated with antibodies against FAK (#05‐182; Millipore, Marousi, Athens, Greece), pAkt (#4060; Cell Signaling Technology Inc., Leiden, The Netherlands), Akt (#4691; Cell Signaling Technology Inc.) or tubulin for normalization, followed by detection using enhanced chemiluminescence (GE Healthcare, Chicago, United States).

### 2.4 Mice

All mice were maintained in a pathogen‐free facility at the Medical School of the University of Crete. Experimental procedures were approved by the Research Animal Care Committee of the Medical School, University of Crete, and by the Veterinary Department of Heraklion Prefecture (Protocol No. 54827, 2182), in accordance with national and EU regulations. NOD.*Cg‐Prkdc* ^*scid*^ *Il2rg* ^*tm1Wjl*^ (NOD *scid* gamma or NSG) mice were obtained from Taconic Biosciences (Leverkusen, Germany) A/S and from the Institute of Molecular Biology and Biotechnology, Crete, Greece. BALB/c nude male mice were obtained from Charles River Laboratories (Wilmington, US‐DE, US). δ^D910A/D910A^ mice were kindly provided by Prof. Bart Vanhaesebroeck (UCL).

Group sample sizes were determined based on calculations using the NC3Rs‐recommended Resource Equation method, pilot experiments, and prior knowledge of the variability of breast and melanoma tumors in NSG and BALB/c nude mice respectively [22, 23, 24]. All mice used were female or male (where indicated), age‐matched (2–3 months), and in good health. Animals were randomly assigned to experimental groups at the start of each experiment. Following tumor cell injections, an equal number of mice were randomly allocated to each treatment arm. Each experiment included a minimum of 7 mice per group (specific numbers are indicated in the figure legends) and was independently repeated at least three times to assess reproducibility.

### 2.5 *In vivo* studies of tumor growth in xenograft models

The xenograft procedures and pharmacological treatment protocols used in this study were based on methods described previously [22, 23, 24], with the experimental conditions specific to the present study detailed below. Anesthetized female or, where indicated male, NSG mice were inoculated subcutaneously into the right mammary fat pad on day 0 with 1 × 10^6^ MDA‐MB‐231 cells or B11 or D1 clone of MDA‐MB‐231 cells or, where indicated with 5 × 10^5^ B16.F10 melanoma cells in 100 μL PBS. When this is indicated, the PI3K p110δ inhibitor IC87114 (35 mg·kg^−1^) was administered in B16 tumor‐bearing Balb/c nude mice by oral gavage once daily [23] alone or in combination with the FAK inhibitor PF573,228 (PF‐228) at a suboptimal dose of 12.5 mg·kg^−1^ PF573228 [17]. Tumor growth was monitored by measurement of the longest perpendicular tumor diameters using a digital caliper every 3–6 days. The tumor volume (V) was calculated using the formula V (mm^3^) = (length (mm) × width (mm)^2^) × 0.5 [25, 26]. Mouse body weight was monitored weekly. At the end of the study, animals were euthanized, and primary tumors were collected for the experiments described below.

### 2.6 Intratumoral moderate suppression of FAK expression by siRNA in mice

The siRNA targeting the *PTK2* gene (pp125FAK) was obtained from Dharmacon (Waltham, MA, US) (ON‐TARGET plus SMART pool Human PTK2 siRNA, #L‐003164). Intratumoral siRNA administration was performed using an approach described previously [22, 23, 27], with the FAK/PTK2‐specific conditions used in the present study detailed below. The siRNA was complexed with Oligofectamine™ Transfection Reagent (#12252011; Invitrogen) and administered by intratumoral injections starting on day +10 when the tumor was palpable. Intratumoral PTK2 siRNA injections were performed every other day, whereas control groups were receiving intratumoral injections of vehicle alone. Pilot experiments testing varying amounts of *PTK2* siRNA in MDA‐MB‐231‐bearing tumor mice that were performed revealed that intratumoral injections of 0.7–0.8 nmoles of siRNA per tumor give a moderate suppression of FAK expression.

### 2.7 Adoptive macrophage transfer

The adoptive macrophage transfer procedure was performed as previously described [22, 23], with macrophage isolation based on the method described in [28]. Wild‐type (WT) mice or δ^D910A/D910A^ mice in which p110δ alleles are replaced by a kinase‐dead version of p110δ mutated in the ATP binding site [29], were served as macrophage donor mice. WT and δ^D910A/D910A^ mice received intraperitoneal injections of 5 mL of 3% Brewer thioglycollate medium and 1 week later were euthanized and peritoneal macrophages were collected [28]. On day 15, 5 × 10^5^ macrophages from WT or δ^D910A/D910A^ mice were intravenously administered into tumor‐bearing NSG mice. The adoptive macrophage transfer was repeated every 48 h [22, 23]. Tumor growth was monitored and calculated as described above.

### 2.8 Tumor cell blood burden

The tumor cell blood burden was assessed as previously described [22, 23, 24, 30]. Mice were anesthetized, and blood was collected from the right atrium using a heparin‐coated 25‐gauge syringe. Samples were plated in culture dishes with Geneticin to selectively grow tumor cells, and blood burden was calculated as the number of colonies per volume of blood.

### 2.9 BrdU incorporation

BrdU incorporation was assessed using a procedure described previously [22, 23, 24], with the experimental and quantification conditions used in the present study specified below. To assess the proliferative activity of tumor cells, a BrdU staining kit (#2760; Millipore) was used following the manufacturer's protocol. In brief, mice were administered 100 mg·kg^−1^ body weight of 5‐bromo‐2′‐deoxyuridine (BrdU; Calbiochem) via intraperitoneal injection 2 h prior to euthanasia, and BrdU‐positive tumor cells were subsequently detected. The quantification of BrdU‐positive cells was performed using imagej software (NIH). Data are expressed as means ± s.e.m. of BrdU‐positive cells relative to hematoxylin‐stained cells, counted from 5 to 8 randomly selected fields per section across 3 sections per measurement. BrdU incorporation assays were conducted on tissues from at least three independent experimental groups for each treatment condition, with consistent results observed across replicates.

### 2.10 TUNEL assay

Tumor cell apoptosis was assessed by TUNEL using a procedure described previously [22, 23, 24], with the experimental and quantification conditions used in the present study specified below. Apoptosis in tumor cells was detected using the DeadEnd Colorimetric TUNEL System (#G7130; Promega, Madison, WI, US) according to the manufacturer's instructions. TUNEL‐positive cells were quantified using imagej software (NIH). Data are expressed as means ± s.e.m. of TUNEL‐positive cells relative to hematoxylin‐stained cells, counted from 5 to 8 randomly selected fields per section across 3 sections per measurement. The TUNEL assay was performed on tissues from at least three independent experimental groups of animals for each treatment condition, and comparable results were obtained across experiments.

### 2.11 Statistical analysis

Error bars displayed in the Figure section represent SEM or SD and were calculated from technical or biological replicates as described in the figure legends and in the description of the respective methods. Data shown are representative of at least 3 independent experiments, including animal studies, histological images, blots, and gels. Data were analyzed using the STATISTICA 7 statistical software package (RRID:SCR_014213). Statistical significance was determined using the non‐parametric Mann–Whitney test; *P < 0.05; **P < 0.01; ***P < 0.001.

## 3 Results

### 3.1 Impact of differential FAK expression on breast cancer progression

**Fig. 1:**
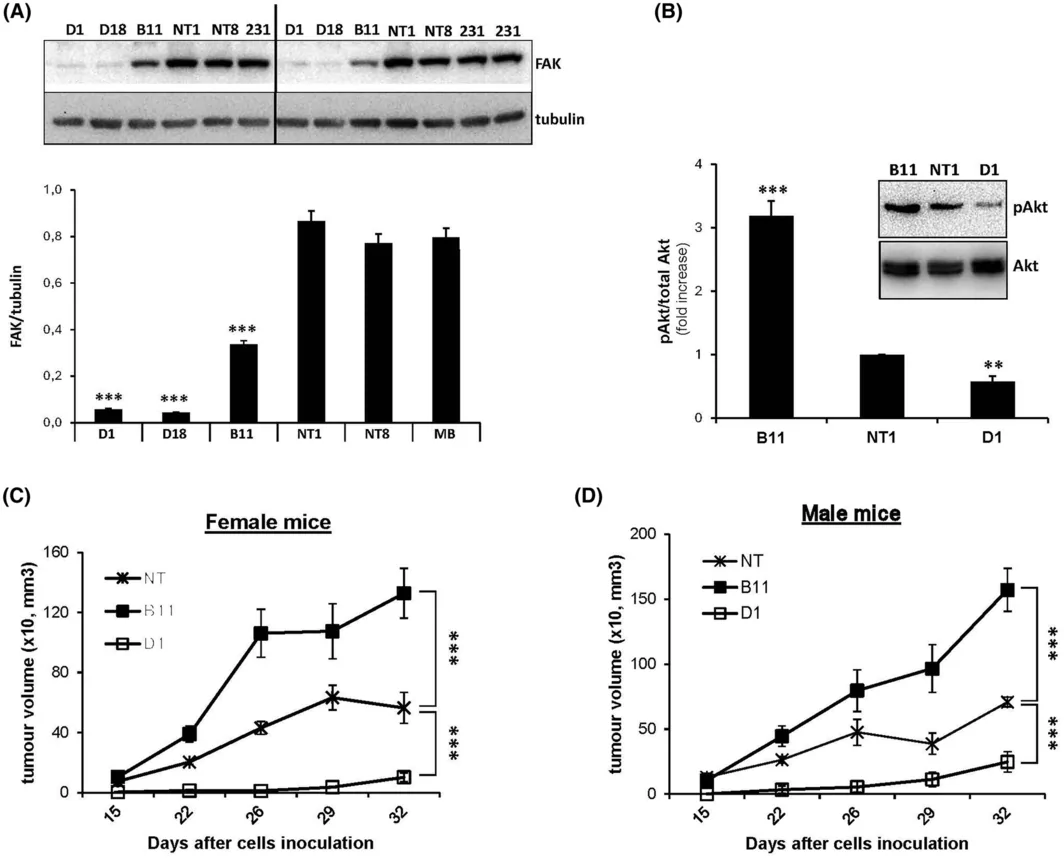
Differential FAK suppression produces opposing effects on Akt activation and breast tumor growth. (A) MDA‐MB‐231 cells were stably transfected with different focal adhesion kinase (FAK) short hairpin RNAs (shRNAs), generating multiple clonal cell lines with varying degrees of FAK silencing or with non‐targeting (NT) control shRNA. Quantification of FAK expression in representative MDA‐MB‐231‐derived clones in two representative western blots is shown. Tubulin was used as a loading control. Graphs represent the mean ± standard error of the mean (SEM) from three independent experiments. (B) Representative clones exhibiting moderate (B11) or strong (D1) FAK knockdown were selected for further analysis. Phosphorylation of Akt in B11, NT1, and D1 cells, representing moderate FAK silencing, normal FAK expression, and strong FAK silencing, respectively, was analyzed by western blot (WB) analysis for phosphorylated Akt (pAkt; Ser473) and total Akt. The ratio of phosphorylated to total Akt was quantified. Graphs represent the mean ± SEM from three independent experiments. (C, D) Tumor growth of NT, B11, and D1 cells following inoculation into female (C) or male (D) NOD scid gamma (NSG) mice (*n* = 9 mice/group). All graphs represent means ± SEM. Statistically significant differences between each group and NT1 are indicated by **P < 0.01 or ***P < 0.001, as determined by the Mann–Whitney test.

To investigate the impact of differential FAK expression levels on breast cancer progression, we generated MDA‐MB‐231 human breast cancer cells stably transfected with shRNA vectors targeting FAK. This approach yielded multiple clonal cell lines with varying degrees of silencing efficiency, allowing us to examine the biological consequences of differential FAK expression. Two representative clones were selected: one exhibiting strong FAK silencing, designated D1, and one exhibiting moderate FAK silencing, designated B11 (Fig. 1A). Clones obtained after transfection with the non‐targeting shRNA control vector (NT) expressed FAK at levels comparable to those observed in untransfected MDA‐MB‐231 cells (Fig. 1A). We then examined Akt phosphorylation levels in clones B11, D1, and NT1 and found that D1 cells displayed reduced Akt phosphorylation, whereas B11 cells exhibited markedly increased Akt phosphorylation compared with NT1 cells (Fig. 1B). These findings suggest that moderate FAK suppression, but not strong FAK silencing, enhances Akt signaling.

We next investigated whether different levels of FAK expression in breast cancer cells have distinct effects on tumor progression *in vivo*. To this end, breast tumors were generated in female NSG mice using either control NT cells or the D1 and B11 clones. B11 cells formed rapidly expanding tumors within weeks, whereas D1‐derived tumors were absent or minimal (Fig. 1C). A similar pattern was observed in male mice, suggesting that the effect of moderate FAK expression on tumor growth is independent of sex (Fig. 1D).

**Fig. 2:**
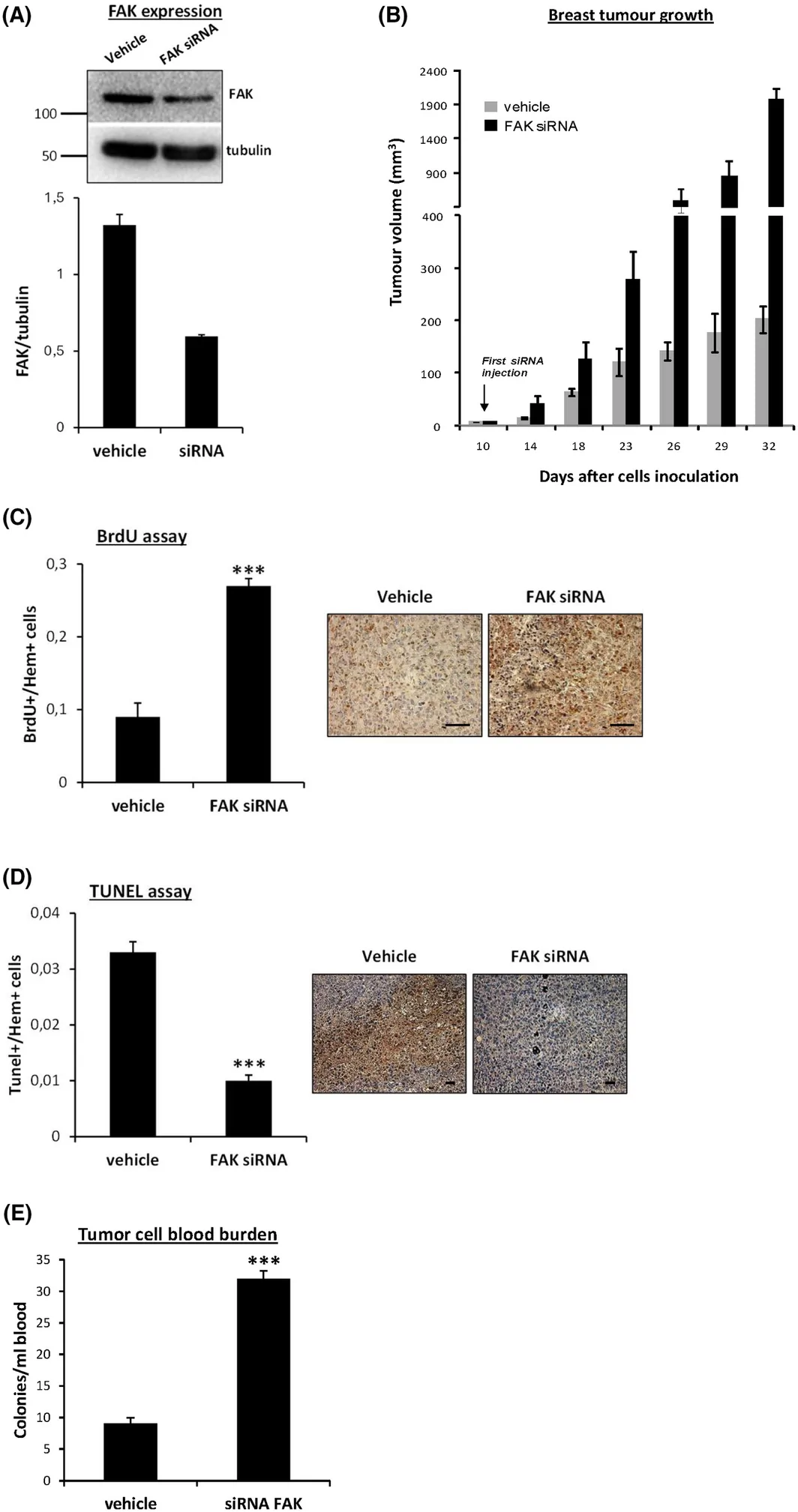
Intratumoral administration of FAK/PTK2 siRNA at a dose that induces moderate FAK suppression accelerates breast tumor progression. (A) Western blot analysis of focal adhesion kinase (FAK; encoded by *PTK2*) expression in tumors treated with vehicle or FAK/PTK2 small interfering RNA (siRNA). Tubulin was used as loading control. The blot shown is representative of three independent experiments. (B) MDA‐MB‐231 breast tumor growth following intratumoral administration of vehicle or FAK/PTK2 siRNA. The timing of the first siRNA injection is indicated. *n* = 7 mice/group. The experiment was independently repeated three times with comparable results. (C) Cell proliferation in tumors excised from mice that were treated with intratumoral injections of FAK/PTK2 siRNA or vehicle was determined by incorporation of 5‐bromo‐2′‐deoxyuridine (BrdU). Representative BrdU‐stained (brown spots) tumor sections are shown in the right panels. Scale bar = 50 μm. Quantification of BrdU‐positive cells relative to hematoxylin‐stained cells is shown in the left panel. *n* = 7 mice/group. (D) Apoptosis in tumors excised from mice that were treated with intratumoral injections of FAK/PTK2 siRNA or vehicle was determined by terminal deoxynucleotidyl transferase dUTP nick‐end labeling (TUNEL). Representative TUNEL‐stained (brown spots) tumor sections are shown in the right panels. Scale bar = 50 μm. Quantification of TUNEL‐positive cells relative to hematoxylin‐stained cells is shown in the left panel. *n* = 7 mice/group. (E) Tumor cell blood burden at the experimental endpoint was used as a measure of tumor cell intravasation in mice receiving intratumoral FAK/PTK2 siRNA or vehicle. *n* = 7 mice/group. Data are presented as mean ± standard error of the mean (SEM). Statistical significance in (C–E) was determined using the Mann–Whitney test; ***P < 0.001. The data shown are representative of three independent experiments.

To further confirm this effect, we assessed the impact of FAK/PTK2 siRNA at a concentration that results in moderate suppression of FAK expression (Fig. 2A) in MDA‐MB‐231 breast tumors grown in NSG mice. Indeed, intratumoral administration of FAK siRNA rapidly accelerated tumor growth (Fig. 2B) and increased tumor cell proliferation (Fig. 2C), whereas apoptosis was significantly reduced in tumor cells (Fig. 2D). Moreover, the number of tumor cells detected in the blood, which is a direct evaluation of intravasation and therefore of efficiency of metastasis [30], was found to be significantly increased in mice bearing tumors treated with FAK siRNA compared with those injected with vehicle (Fig. 2E). These findings indicate that moderate suppression of FAK expression enhances tumor growth and metastatic potential.

Together, the above results strongly suggest that moderate FAK silencing accelerates breast tumor progression, in contrast to strong FAK silencing, which suppresses tumor growth.

### 3.2 Inactivation of p110δ PI3K in macrophages counteracts tumor progression induced by moderate FAK suppression in breast and melanoma tumors

We next aimed to determine whether p110δ PI3K inactivation in macrophages could counteract breast tumor growth induced by moderate FAK silencing. Since MDA‐MB‐231 cells express high levels of p110δ and p110δ inhibition directly affects tumor cell signaling, including FAK activity [18, 31], systemic administration of a p110δ inhibitor would not allow the macrophage‐specific contribution to be distinguished. We therefore used macrophages with genetically inactivated p110δ, an approach previously shown to be sufficient to reduce breast tumor growth and metastasis [22].

**Fig. 3:**
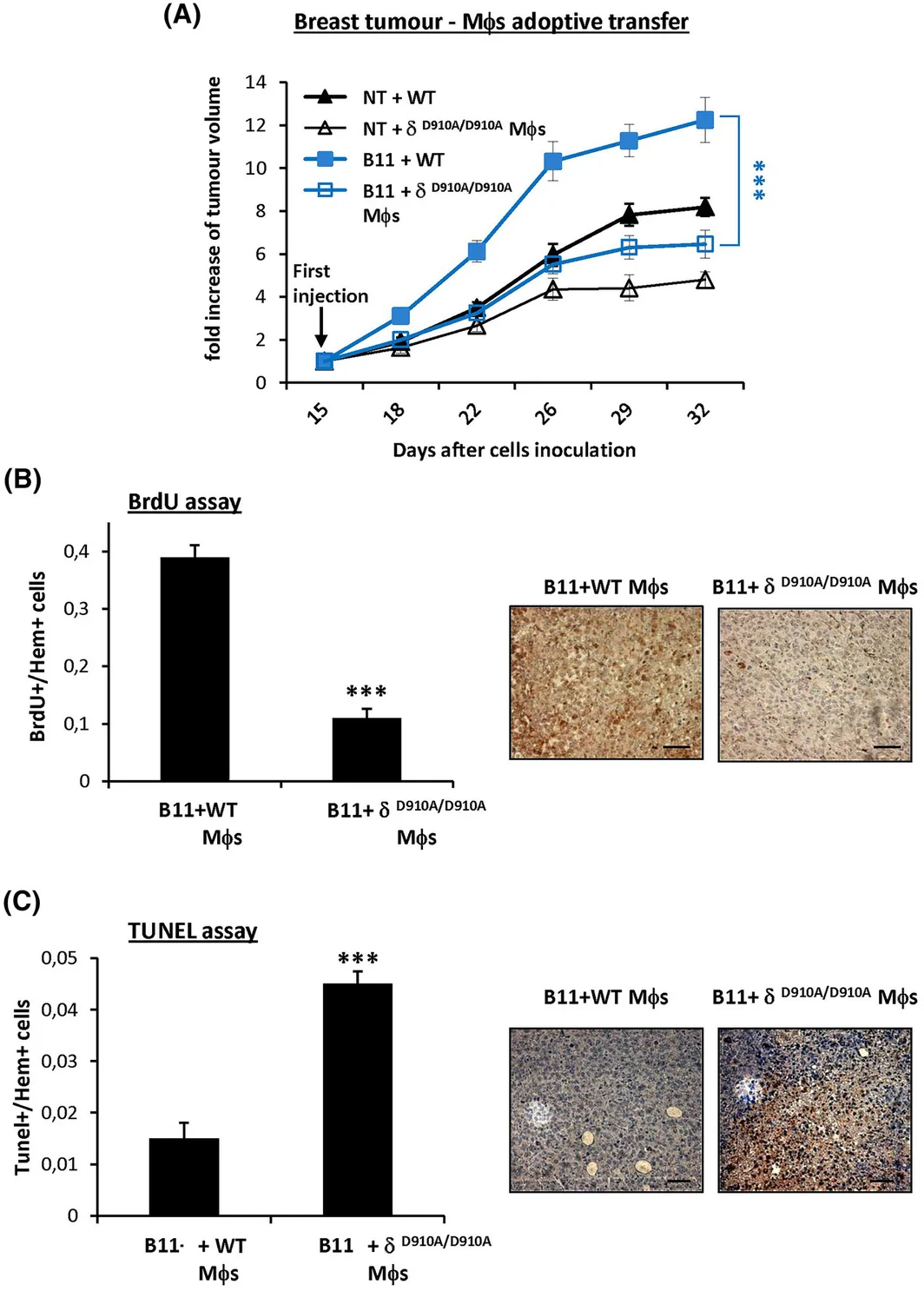
Macrophage p110δ phosphoinositide 3‐kinase inactivation counteracts breast tumor progression induced by moderate FAK suppression. (A) NOD scid gamma (NSG) mice were inoculated with either non‐targeting control (NT) or moderately FAK‐silenced B11 MDA‐MB‐231 cells on day 0 and treated with intravenous injections of wild‐type (WT) or p110δ kinase‐inactive δ^D910A/D910A^ macrophages from day 15 and every other day thereafter until the end of the experiment. Tumor growth was monitored. *n* = 8 mice/group. (B) Cell proliferation in tumors excised from B11 tumor‐bearing mice receiving WT or δ^D910A/D910A^ macrophages was determined by 5‐bromo‐2′‐deoxyuridine (BrdU) incorporation. Representative BrdU‐stained (brown spots) sections are shown in the right panels. Scale bar = 50 μm. Quantification is shown in the left panel. *n* = 8 mice/group. (C) Apoptosis in tumors excised from B11 tumor‐bearing mice that were treated with intravenous injections of WT or δ^D910A/D910A^ macrophages was determined by terminal deoxynucleotidyl transferase dUTP nick‐end labeling (TUNEL). Representative TUNEL‐stained (brown spots) tumor sections are shown in the right panels. Scale bar = 50 μm. Quantification is shown in the left panel. *n* = 8 mice/group. Data are presented as mean ± standard error of the mean (SEM). Statistical significance was determined using the Mann–Whitney test; ***P < 0.001. The experiments were independently repeated three times with comparable results.

We therefore carried out adoptive macrophage transfer experiments in NSG mice that have defective macrophages bearing MDA‐MB‐231‐derived NT or B11 tumors. Interestingly, when δ^D910A/D910A^ macrophages, which express genetically inactivated p110δ [29] were transferred into B11 tumor‐bearing mice, tumor burden was significantly reduced compared with that observed in B11 tumor‐bearing mice receiving WT macrophages (Fig. 3A). In line with this finding, the number of BrdU‐positive cells was significantly reduced in B11 tumor specimens from mice receiving δ^D910A/D910A^ macrophages compared with those from B11 tumor‐bearing mice receiving WT macrophages (Fig. 3B), indicating reduced tumor cell proliferation. Conversely, the number of TUNEL‐positive cells was significantly increased in B11 tumor‐bearing mice receiving δ^D910A/D910A^ macrophages (Fig. 3C), suggesting enhanced apoptosis in tumor cells.

Previous studies have shown increased melanoma tumor growth in mice treated with a suboptimal concentration of the FAK inhibitor PF‐228 [17]. We therefore investigated whether p110δ inhibition could also counteract melanoma tumor growth induced by incomplete pharmacological inhibition of FAK. To this end, B16 melanoma tumors were generated in Balb/c nude mice, which retain functional macrophages, and mice were treated orally with the FAK inhibitor PF‐228 at suboptimal concentration [17], either alone or in combination with the selective p110δ inhibitor IC87114 at an optimal concentration [22, 23]. This approach was based on our previous finding that pharmacological inhibition of p110δ PI3K suppresses melanoma tumor growth predominantly by targeting macrophages, without exerting a substantial direct effect on melanoma cells [23] Thus, the melanoma treatment model is conceptually analogous to the macrophage‐targeted p110δ inactivation approach used in the breast tumor model (Fig. 3A).

**Fig. 4:**
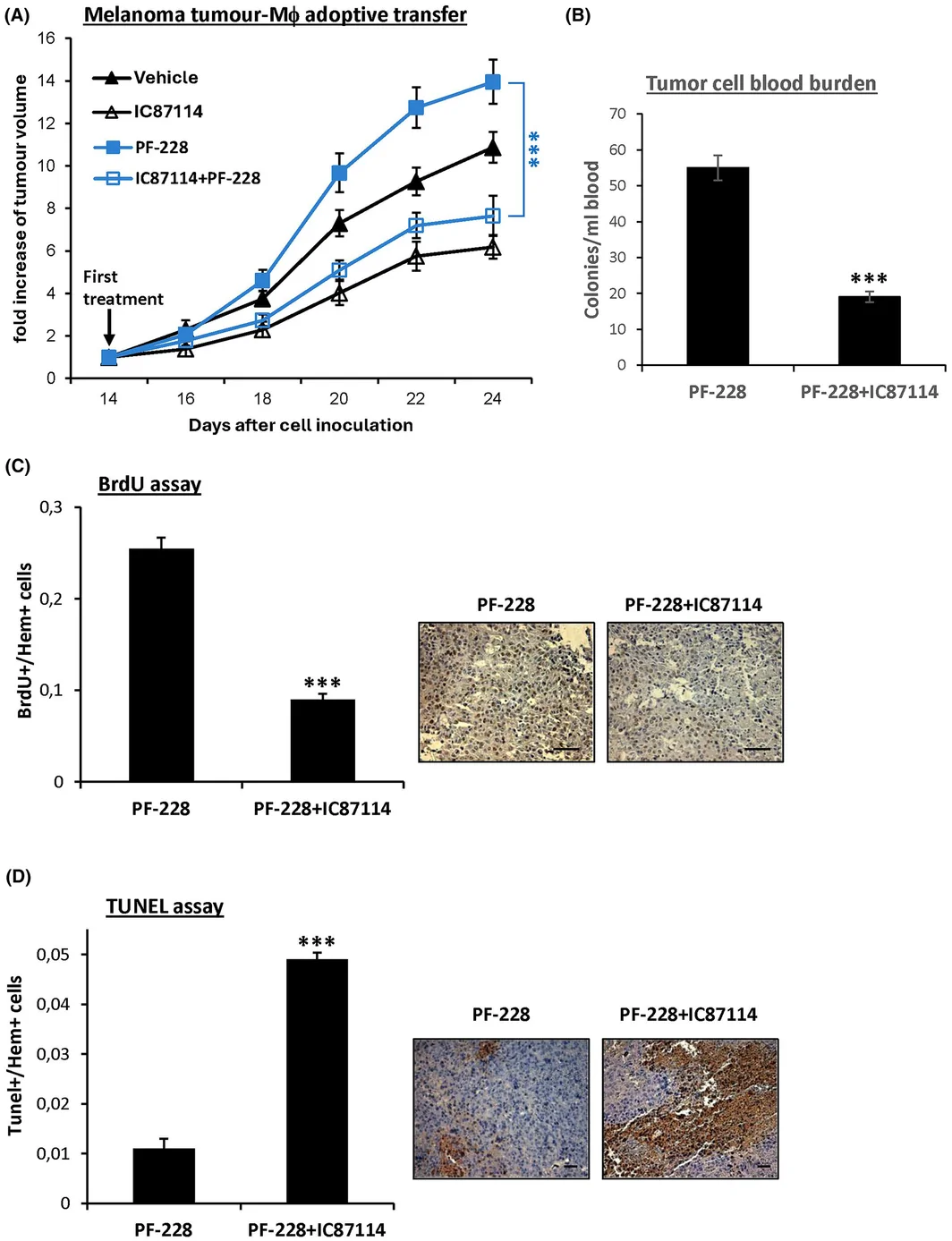
p110δ phosphoinositide 3‐kinase inhibition prevents melanoma tumor progression induced by suboptimal focal adhesion kinase inhibition. (A) Balb/c nude mice were inoculated with B16 melanoma cells on day 0 and treated once daily by oral gavage from day +14 with vehicle, the selective p110δ phosphoinositide 3‐kinase (PI3K) inhibitor IC87114 (35 mg·kg^−1^), the focal adhesion kinase (FAK) inhibitor PF‐228 (12.5 mg·kg^−1^), or the combination of IC87114 and PF‐228. Tumor growth was monitored. *n* = 8 mice/group for vehicle‐, IC87114‐ and PF‐228‐treated mice. *n* = 9 mice/group for combination‐treated mice. (B) Intravasation efficiency of cancer cells as determined by tumor cells blood burden at the end point of the experiment in mice which received PF‐228 or combination of IC87114 and PF‐228. *n* = 8 mice/group. (C) Cell proliferation in tumors excised from B16 melanoma tumor‐bearing mice that were treated with PF‐228 or combination of IC87114 and PF‐228 was determined by 5‐bromo‐2′‐deoxyuridine (BrdU) incorporation. Representative BrdU‐stained (brown spots) sections are shown in the right panels. Scale bar = 50 μm. Quantification is shown in the left panel. *n* = 8 mice/group. (D) Apoptosis in tumors excised from B16 melanoma tumor‐bearing mice that were treated with PF‐228 or combination of IC87114 and PF‐228 was determined by terminal deoxynucleotidyl transferase dUTP nick‐end labeling (TUNEL). Representative TUNEL‐stained (brown spots) tumor sections are shown in the right panels. Scale bar = 50 μm. Quantification is shown in the left panel. *n* = 8 mice/group. Data are presented as mean ± standard error of the mean (SEM). Statistical significance was determined using the Mann–Whitney test; ***P < 0.001. The experiments were independently repeated three times with comparable results.

As expected, low‐dose PF‐228 increased tumor burden compared with vehicle‐treated mice (Fig. 4A). However, combined treatment with PF‐228 and IC87114 significantly prevented this increase in tumor burden, reducing it to levels comparable to those observed in mice treated with the p110δ inhibitor alone (Fig. 4A). We then examined the effects of the combined treatment on melanoma tumor cell metastatic potential, proliferation, and apoptosis. Combined inhibition of FAK and p110δ significantly reduced tumor cell burden in the blood (Fig. 4B), indicating effective suppression of spontaneous intravasation and dissemination of melanoma cells. Moreover, the number of BrdU‐positive cells was significantly reduced in tumor specimens from mice receiving both inhibitors compared with those receiving the FAK inhibitor alone (Fig. 4C), indicating reduced tumor cell proliferation. In contrast, the number of TUNEL‐positive cells was significantly increased in mice treated with PF‐228 and IC87114 (Fig. 4D), suggesting enhanced tumor cell apoptosis.

Together, these findings demonstrate that inactivation of p110δ PI3K in macrophages counteracts the tumor‐promoting effects induced by moderate FAK suppression or incomplete FAK inhibition. This effect was observed in both breast and melanoma tumor models and was associated with reduced tumor burden, decreased tumor cell proliferation, enhanced apoptosis, and diminished tumor cell intravasation/dissemination. These results suggest that macrophage‐targeted p110δ PI3K inactivation may represent a complementary therapeutic strategy to prevent the adverse tumor‐promoting consequences of partial FAK inhibition.

## 4 Discussion

FAK is widely recognized as a central regulator of tumor cell adhesion, migration, proliferation, survival, invasion, and communication with the tumor microenvironment [4, 6, 9]. Consequently, FAK inhibition has been proposed as a promising therapeutic strategy in several cancer types, with pharmacological approaches targeting either FAK kinase activity or kinase‐independent scaffold functions currently under preclinical and clinical investigation [6, 9, 12]. However, the present study demonstrates that the biological outcome of FAK targeting is highly dependent on the extent of FAK suppression. Whereas strong FAK silencing impaired breast tumor growth, moderate FAK suppression accelerated tumor progression. These findings suggest that incomplete inhibition of FAK may not simply produce a weaker anti‐tumor effect but may instead trigger tumor‐promoting signaling and enhance malignant behavior.

Using MDA‐MB‐231 breast cancer cells with different degrees of FAK silencing, we found that strong FAK suppression reduced Akt phosphorylation and markedly impaired tumor formation *in vivo*. In contrast, moderate FAK silencing increased Akt phosphorylation and promoted rapid tumor growth. This indicates that different residual levels of FAK expression can generate distinct, and even opposing, biological responses. Since the PI3K/Akt pathway is a major regulator of tumor cell survival, proliferation, and resistance to apoptosis [4, 7, 8], the increased Akt activation observed in moderately FAK‐silenced cells may provide a survival and proliferative advantage, thereby contributing to the aggressive tumor phenotype. Importantly, the tumor‐promoting effect of moderate FAK suppression was observed in both female and male mice, suggesting that this response is independent of sex.

The pro‐tumorigenic effect of moderate FAK suppression was further confirmed using intratumoral administration of FAK/PTK2 siRNA at a dose that partially reduced FAK expression. This approach avoided reliance solely on stable clonal cell lines and supported the conclusion that moderate FAK suppression itself can accelerate tumor progression. In this setting, FAK siRNA administration increased tumor growth and tumor cell proliferation, while reducing apoptosis. Moreover, the number of tumor cells detected in the blood was significantly increased, suggesting enhanced intravasation and dissemination potential. These findings indicate that partial FAK suppression promotes multiple features associated with tumor aggressiveness, including enhanced growth, increased proliferation, reduced apoptosis, and increased tumor cell entry into the circulation.

These results have important therapeutic implications. Pharmacological inhibitors targeting FAK kinase activity, as well as approaches designed to interfere with FAK scaffold functions, are being evaluated in preclinical and clinical settings [4, 9, 12]. However, complete and sustained pharmacological inhibition of FAK may be difficult to maintain *in vivo* because of pharmacokinetic variability, heterogeneous drug penetration, tumor cell heterogeneity, and adaptive signaling responses. Our findings raise the possibility that suboptimal or incomplete FAK inhibition could, under certain conditions, generate adverse tumor‐promoting effects. This does not argue against FAK as a therapeutic target, but rather suggests that the degree, duration, and cellular context of FAK inhibition are critical determinants of therapeutic outcome. This concept is consistent with the increasing view that FAK‐targeted therapies may be most effective when used in rational combinations rather than as isolated interventions [9].

A key finding of this study is that macrophage p110δ PI3K inactivation counteracted the tumor‐promoting effect induced by moderate FAK suppression. Transfer of p110δ‐inactivated macrophages significantly reduced tumor burden, decreased tumor cell proliferation, and increased apoptosis compared with transfer of WT macrophages. This is consistent with our previous study demonstrating that systemic p110δ inhibition markedly suppressed MDA‐MB‐231 xenograft growth, while selective genetic inactivation of p110δ in macrophages was itself sufficient to reduce tumor growth, tumor‐associated macrophage accumulation, and metastatic dissemination [22]. These findings indicate that the activation of FAK induced by p110δ inhibition in breast cancer cells [18, 31] does not override the overall antitumor effect of systemic p110δ blockade, which likely reflects its combined actions on tumor cells and the macrophage compartment. From a therapeutic perspective, combined systemic FAK and p110δ inhibition could therefore be beneficial, provided that FAK activity is inhibited sufficiently. Effective FAK blockade could prevent the compensatory FAK activation induced by p110δ inhibition, while p110δ blockade would simultaneously exert direct antitumor effects and restrict macrophage recruitment and tumor‐supporting activity. Conversely, the present findings demonstrate that moderate or incomplete FAK suppression can promote tumor growth and could therefore attenuate or complicate the benefit of systemic p110δ inhibition. Thus, although macrophage‐specific p110δ inactivation was used here to define the contribution of this cellular compartment, the net therapeutic effect of combined systemic FAK and p110δ inhibition is likely to depend critically on the extent and duration of FAK target inhibition and requires direct experimental evaluation.

The melanoma model further supported this concept in a pharmacological setting. Previous work showed that suboptimal concentrations of the FAK inhibitor PF‐228 increase melanoma tumor growth [17]. Consistent with this, low‐dose PF‐228 enhanced melanoma tumor burden in our experimental model. However, combined treatment with PF‐228 and the p110δ inhibitor IC87114 prevented this increase, reducing tumor burden to levels comparable to those observed with p110δ inhibition alone. In addition, combined FAK and p110δ inhibition reduced circulating melanoma tumor cells, decreased tumor cell proliferation, and increased apoptosis. These findings suggest that p110δ inhibition can counteract the adverse effects of incomplete FAK inhibition not only in breast tumors, where macrophage p110δ was genetically targeted, but also in melanoma tumors treated pharmacologically. Given that PF‐228 targets FAK catalytic activity [32], these results further support the concept that incomplete pharmacological inhibition of FAK kinase activity can have biological effects distinct from those caused by strong FAK suppression.

Together, the breast and melanoma tumor models support a common principle: Partial suppression of FAK activity or expression can promote tumor progression, whereas p110δ PI3K inactivation, particularly in macrophages, can oppose this effect. This suggests that macrophages are important mediators of the tumor response to incomplete FAK inhibition. Since p110δ is highly enriched in leukocytes and has a major role in immune cell signaling [29, 33, 34], its inhibition may alter macrophage recruitment, localization, polarization, or tumor‐supportive functions. In this context, macrophage p110δ activity may contribute to a microenvironment that supports tumor cell proliferation, survival, and intravasation under conditions of partial FAK suppression. Therefore, targeting macrophage p110δ may represent a complementary strategy to prevent or reverse tumor‐promoting consequences of incomplete FAK inhibition.

An important aspect of this work is that FAK suppression produced divergent effects depending on its extent. This may help explain why therapeutic targeting of signaling proteins can produce variable outcomes *in vivo*. Complete inhibition of a pro‐tumorigenic pathway may suppress tumor growth, whereas partial inhibition may relieve specific regulatory constraints while preserving or activating compensatory survival pathways. In the case of FAK, moderate suppression appears to favor Akt activation, increased proliferation, reduced apoptosis, and enhanced tumor cell dissemination. These findings emphasize the need to evaluate not only whether a target is inhibited, but also the degree and duration of inhibition achieved *in vivo*. This may be particularly important for FAK, which has both kinase‐dependent and scaffold‐dependent functions and acts in tumor cells as well as in stromal and immune compartments [6, 9].

## 5 Conclusion

Our findings demonstrate that the extent of FAK suppression is a critical determinant of tumor outcome. Whereas strong FAK suppression inhibits tumor growth, partial or incomplete FAK inhibition can exert tumor‐promoting effects. Macrophage p110δ PI3K inactivation counteracts these adverse effects, supporting its potential as a complementary therapeutic strategy when complete and sustained FAK inhibition cannot be achieved. These findings highlight the importance of considering both the degree of target inhibition and tumor–immune cell interactions when designing FAK‐targeted therapeutic approaches.

## Conflict of interest

The authors declare no conflict of interest.

## Author contributions

LX, NT and AT performed experiments and data analyses. MT performed histology and interpreted histopathology and immunohistochemistry. EAP conceived the project, designed and analyzed the experiments, supervised experiments and wrote the paper.

## Data Availability

The authors declare that the data supporting the findings of this study are available within the paper and/or are available upon request from the corresponding author.
